# The mobile surgical outreach program for management of patients with genital fistula in the Democratic Republic of Congo

**DOI:** 10.1002/ijgo.13036

**Published:** 2020-01-13

**Authors:** Raha Maroyi, Laura Keyser, Lauren Hosterman, Amisi Notia, Denis Mukwege

**Affiliations:** ^1^ Department of Urogynecology Panzi General Referral Hospital Bukavu Democratic Republic of Congo; ^2^ Faculty of Medicine Evangelical University in Africa Bukavu Democratic Republic of Congo; ^3^ Mama, LLC Canton MA USA

**Keywords:** Community outreach, Democratic Republic of Congo, Genital fistula, Mobile surgical outreach, Surgical training

## Abstract

**Objective:**

To describe components of the mobile surgical outreach (MSO) program as a model of care delivery for women with genital fistula; present program results; and discuss operational strengths and challenges.

**Methods:**

A retrospective observational study of routinely collected health data from women treated via the MSO program (2013–2018). The program was developed at Panzi Hospital in the Democratic Republic of Congo to meet the needs of women with fistula living in remote provinces, where travel is prohibited. It includes healthcare delivery, medico‐surgical training, and community sensitization components.

**Results:**

The MSO team cared for 1517 women at 41 clinic sites across 18 provinces over the study period. Average age at presentation was 31 years (range, 1–81 years). Most women (n=1359, 89.6%) presented with vesicovaginal fistula. Most surgeries were successful, and few women reported residual incontinence postoperatively. Local teams were receptive and engaged in clinical skills training and public health education efforts.

**Conclusion:**

The MSO program addresses the backlog of patients awaiting fistula surgery and provides a template for a national strategic plan to treat and ultimately end fistula in DRC. It offers a patient‐centered approach that brings medico‐surgical care and psychosocial support to women with fistula in their own communities.

## Abstract 2

Panzi Hospital's mobile surgical outreach program provides fistula care to women in their communities and represents a scalable model for treatment and prevention of fistula.

## 1 INTRODUCTION

Female genital fistula is often a direct result of inadequate obstetric health services for women with obstructed labor; congenital, iatrogenic, and genital trauma represent other less frequent causes.^1^, ^2^, ^3^ Women with fistula suffer physical, socioeconomic, and psychological consequences and experience a high level of disability, which further limits their access to adequate health services and skilled surgical care.^4^, ^5^, ^6^ The Democratic Republic of Congo (DRC) is a country characterized by weak maternal health indicators; the World Bank reports a fertility rate of 6 births per woman and a maternal mortality ratio of 550 per 100 000 live births.^7^ Additionally, the vast size of the DRC, ongoing insecurity, extreme poverty, and poor infrastructure further compound barriers to health care.^8^ Despite efforts to improve obstetric care, many women lack access to sufficient perinatal care and suffer complications from childbirth, including genital fistula.^7^

It is difficult to estimate the incidence and prevalence of fistula in DRC as the majority of the country's 77 million people live in rural areas that are difficult to reach and significantly underdeveloped.^9^ Political instability, conflict, and inadequate investment in health infrastructure further contribute to this lack of data. In 2018, the DRC's Ministry of Health reported 42 000 women with genital fistula are awaiting surgical care.^10^ However, a national strategic policy for fistula prevention and treatment does not exist to address this significant burden.^3^ As a result, women with fistula often suffer for years with the associated primary and secondary health consequences while waiting for surgical care.^5^, ^11^

The Panzi General Reference Hospital in eastern DRC was established in 1999 as a tertiary care facility with specialization in obstetrics and gynecology. Since its inception, thousands of women have received surgical care for repair of gynecologic injuries, including fistula.^12^ In 2011, Panzi Hospital developed a surgical outreach program after receiving two women who had walked over 1000 km to receive care. Recognizing geographical barriers and persistent insecurity in much of the country, hospital staff worked to create mobile surgical teams to bring skilled health services to patients living in remote areas, so that other women might avoid making such an arduous and potentially life‐threatening journey to reach the hospital.

The objectives of the present paper are to describe the components of the Panzi Hospital mobile surgical outreach (MSO) program as a model of care delivery for women with fistula; to present data highlighting the program's scope and clinical impact; and to discuss operational strengths and challenges to program sustainability and expansion.

## 2 MATERIALS AND METHODS

This paper provides a retrospective descriptive study of programmatic information across MSO sites. The Ministry of Health and South Kivu Province Ethics Committee approved this program and publication of its description and results.

### 2.1 A model for healthcare delivery for women with fistula

The Panzi Hospital MSO program organizes mobile teams, each consisting of two surgeons, one surgical assistant, one nurse, and one anesthetist. Outreach trips occur annually or biannually for most sites, depending on the volume of cases and available resources. Site selection occurs in a two‐step process: (1) identification of accessible hospitals in strategic locations; and (2) initial visit and site readiness assessment.

#### 2.1.1 Site selection and site readiness

Sites may be identified by Panzi Hospital staff by first considering regional health zones and then surveying general hospitals within each zone to determine the volume of fistula cases in that region, as well as whether the local hospital meets the necessary minimum requirements for site selection (Box 1). Alternatively, sites may contact Panzi Hospital to request the MSO team given the number of fistula cases presenting in a particular area. This may be initiated by the general hospital in the region or by community organizations, such as churches, civil society agencies, or women's networks, who express the need for fistula services. Once a site has been identified, a contractual agreement is signed, which commits the MSO team to provide skilled surgical care, including consultation, surgical supplies, medications, and a 3‐month postoperative follow‐up visit. Panzi Hospital also provides transportation and hygiene kits for each patient. The local reference hospital agrees to participate in community sensitization efforts, to support patient care, and to provide space, including operating theater and beds for women to recover for 2–4 weeks postoperatively.

Box 1 **Minimum necessary requirements for site selection.**

**Surgical capacity**

- Adequate light source
- Adaptable operating table allowing for patient positioning
- Power generator
- Sterilization equipment for surgical materials
- Stock of saline solution for IV fluids

**Patient monitoring**

- Minimum 1 each (doctor, nurse) to assist in operating theater and participate in skills training
- Minimum 2 nurses dedicated to postoperative monitoring (day/night shifts)
- One nurse dedicated to translating patient medical histories
- Capacity to hold 50 patients (beds)

**Local emergency committee**

Consists of the following members with well‐defined roles:

- Medical Director: oversees operating theater conditions, manages physician staff
- Nursing Diector: responsible for pre‐ and postoperative nursing activities and scheduling
- Local religious leader and community representative: relay important information to patients, their families, and the community related to outreach activities; responsible for organizing patient meals

**Laboratory**

- Capacity for blood draw and testing
- Blood storage

In advance of the MSO team's arrival, Panzi Hospital sends 1–2 staff to evaluate hospital conditions, including the operating theater and inpatient capacity. This small team also begins a 2–3‐week community sensitization campaign to identify women with fistula and to educate the public about the health services and surgical care provided by the MSO team. This is done collaboratively with the local ministry of health and community leaders. In an effort to reach women living in remote villages, messages are delivered via radio broadcasts, in churches, markets, and other community forums, informing women of the date and location for consultation with the MSO team.

The MSO program includes three key aspects: (1) medico‐surgical care; (2) training of local healthcare teams; and (3) community outreach.

##### 2.1.1.1 Medico‐surgical care

Each woman with fistula receives medico‐surgical care and psychological treatment with the goal of restoring mental and physical well‐being and re‐establishing her role and participation in society. On arrival at the hospital, all women undergo a medical examination by a physician from Panzi Hospital. Fistula diagnosis is confirmed with pelvic examination and a methylene blue dye test. The MSO team utilizes the Panzi score to categorize severity of cases.^13^ Simple fistula cases (score of 0) are scheduled for surgery; complex cases (score 1, 2, or 3) or those with co‐morbidities, such as malnutrition, HIV, or active infection are referred to Panzi Hospital for treatment. Counseling is provided by the physician during the initial consultation, and further supported by trusted community leaders as needed.

Patient consultations are ongoing as patients arrive and include the evaluation and preoperative anesthetic assessment. Conservative management is offered as appropriate. Surgeries are then scheduled each day thereafter for a period of 14–21 days, depending on case volume. Up to 10 surgeries may be performed each day, though on average, the team performs 6–7 surgeries per day for the duration of their visit. Postoperative nursing care is initiated by a member of the MSO team, who works with local nursing staff to ensure each woman is monitored appropriately for complications.

Prior to discharge, each patient receives outpatient counseling regarding sexual activity guidelines, contraception, and future pregnancies. She also receives a summary card of the care received (i.e. fistula type, surgical route, complications, medications, pertinent medical history). This facilitates monitoring of patients at the 3‐month postoperative appointment.

##### 2.1.1.2 Training of local healthcare teams

In an effort toward sustainability, the MSO program involves training of local staff at each site. Initially, the local healthcare teams were brought to Panzi Hospital for training at two separate 3‐month intervals, spaced 3 months apart. In 2016, this approach was modified to better identify local healthcare staff with keen interest and the commitment to learning required for program success and sustainability. The MSO team now provides on‐site training to the local teams over a period of 2–4 weeks, during outreach visits, while simultaneously providing medico‐surgical care to women with fistula. The local teams then complete training at Panzi Hospital for a period of 3 months. This visit is typically scheduled within 3 months after completion of the outreach visit.

The local teams include doctors, laboratory technicians, nurses, midwives, and nurse anesthetists. Training includes prevention of fistula and pre‐ and postoperative care for women undergoing fistula repair. The local surgeon is trained in the repair of simple fistula cases and vaginal prolapse surgery, when resources are available. Training of nurse anesthetists has also become a key component of training, increasing local capacity to provide emergency obstetric services, including safe cesarean deliveries, thus contributing to fistula prevention efforts.

Such training efforts aim to build local capacity to identify and treat women with fistula, as well as those with pelvic organ prolapse, and to encourage referral of complex cases appropriately to Panzi Hospital.

##### 2.1.1.3 Community outreach

Community outreach begins during the site selection process, as outlined above, and continues during the MSO visit. The Panzi Hospital team works alongside local hospital staff to ensure that each patient is educated about her condition and course of treatment. She can also discuss concerns related to her physical and mental health and psychosocial and economic circumstances. Family members are invited to attend educational sessions at the hospital to improve their understanding of causes, treatment, and prevention of fistula.

After the initial MSO visit, community engagement and sensitization responsibilities are transferred from Panzi Hospital to the community. Women who have received treatment from the MSO team often become living testimonials, educating their communities and dispelling myths that fistula is a result of sorcery or witchcraft. The local health ministry, church, and civil society leaders also continue to raise awareness about prevention and treatment of fistula, and the local partner hospital continues to keep a roster of new cases, communicating with Panzi Hospital the volume of cases.

## 3 RESULTS

Records from 2013 to 2018 were available for review. Data available by site varies due to challenges with documentation and data storage, and some information was missing or inconsistent. Efforts were made to reconcile patient information by crosschecking Panzi Hospital and partner site records and addressing discrepancies with local health staff when possible. Results presented here provide a summary of patient characteristics and surgical outcomes.

From 2013 to 2018, the MSO team provided care for 1517 women at 41 clinic sites across 18 provinces. Table 1 presents annual number of cases and patient demographic information. Average age at presentation was 31 years (range, 1–81 years); younger patients (<10 years) largely represent congenital cases or, less frequently, instances of sexualized violence. Most women had little to no formal education and worked as farmers. Table 2 reports clinical characteristics, etiology and type of fistula, and surgical outcomes. Gynecologic and obstetric history were obtained through patient interviews, and thus recall bias and low health literacy may influence responses. Median age at first delivery was 18 years. Approximately 25% of women indicated a subsequent pregnancy after fistula had developed. Most women reported having had at least one previous surgery. Similarly, most women reported understanding fistula as a health problem often related to pregnancy and delivery.

**Table 1:** Number of women treated annually and patient characteristics of the women treated by the mobile surgical outreach team between 2013 and 2018 (n=1517)

	No. (%)
Year treated	n=1503
2013	35 (2.3)
2014	272 (18.1)
2015	208 (13.8)
2016	367 (24.4)
2017	222 (14.8)
2018	399 (26.5)
Average treated per year	251
Age, y	n=1422
Average	31.2
Median	31
Range	1–81
Marital status	n=1409
Married	930 (66.0)
Single	77 (5.5)
Divorced/separated	282 (20.0)
Widowed	120 (8.5)
Education level	n=1431
None	617 (43.1)
Some primary	515 (36.0)
Primary completed	95 (6.6)
Some secondary	166 (11.6)
Secondary or above	38 (2.7)
Profession	n=1407
Farmer	1015 (72.1)
Homemaker	260 (18.5)
Teacher	15 (1.1)
Commerce	46 (3.3)
Other	71 (5.0)

**Table 2:** Type of fistula and surgical outcomes

	No. (%)
Age at first delivery, y	n=1214
Average	16.3
Median	18
Range	0–45
Reported previous operation (n=1339)	774 (57.8)
No. of previous operations	n=774
Average	1.6
Median	1
Range	1–9
Pregnancy after fistula developed (n=1362)	362 (26.6)
Reported history of rape (n=1517)	8 (0.5)
*Current gynecologic health status*	
Understands fistula as a health problem (n=1389)	938 (67.5)
Etiology of fistula	n=1517
Obstetric	1116 (73.6)
Iatrogenic	370 (24.4)
Congenital	23 (1.5)
Associated symptoms	n=1517
Genital irritation	701 (46.2)
Sexual pain	539 (38.3)
Vaginal bleeding	161 (11.2)
Foot drop	287 (19.9)
Type of fistula	n=1517
Vesicovaginal fistula	1359 (89.6)
Rectovaginal fistula	149 (9.8)
Both	39 (2.5)
Surgical outcome	n=1413
Closed fistula	1309 (92.6)
Residual incontinence	30 (2.1)
Failed, open fistula	75 (5.3)

In addition to incontinence, associated symptoms were also reported. Forty‐six percent of women presented with genital irritation due to chronic urine exposure, and 38% complained of sexual pain. The incidence of foot drop reported across sites was high (31.5%). It is the impression of the MSO team that foot drop was not always determined by clinical examination, and patient reports of difficulty walking or lower extremity fatigue may confound this number.

Most women (n=1359, 89.6%) presented with vesicovaginal fistula. Most surgeries successfully closed the fistula, and few women reported residual incontinence, although this was often assessed early postoperatively and may not accurately reflect outcomes at 3 months and beyond. Feedback from patients and local staff has been positive. In addition, Panzi Hospital has built capacity to send four surgical teams on outreach trips at one time.

## 4 DISCUSSION

The Panzi Hospital MSO program has reached women with fistula residing in 18 of the 26 provinces in DRC. While data collection and long‐term follow up remain challenging, program results across 5 years indicate positive surgical outcomes and successful community sensitization campaigns. The program, developed to address many of the barriers to health care faced by women living in DRC, has evolved since its inception, as both areas of strength and opportunities for improvement of care delivery were identified. Box 2 summarizes the strengths and challenges of the MSO program, highlighting areas in need of further development and resource allocation.

Box 2 **Strengths, lessons learned, and challenges of the mobile surgical outreach program.**

**Strengths and lessons learned during program implementation**

Stakeholder involvement

- Panzi Hospital and local health workers, patients and their families, community leaders engaged in program development and implementation

Quality care

- Women report improvement in physical and mental health, are able to return to meaningful family and community life

Health systems strengthening

- Identification of dedicated health staff and training of local providers at affiliate sites improves capacity for comprehensive obstetrics/gynecologic care, including prevention and treatment of fistula
- Specific targeting and uptraining of nursing staff, including nurse anesthetists is essential for program success and sustainability

**Challenges and areas for improvement for future programming**

Resource limitations

- Limited and case‐specific funding for fistula repair only (i.e. no resources for prolapse, other types of UI, fertility, general gynecologic care)
- Lack of family planning resources at local sites
- Inadequate health workforce, few or no physiotherapists, psychologists, other nonphysician health workers to ensure comprehensive, quality care
- No resources for socioeconomic reintegration for women with fistula available at outreach sites
- Limited funds for transportation of complex cases to Panzi Hospital

Poverty‐related challenges

- Poor health literacy of communities
- Limited health‐seeking behavior
- Loss to follow‐up/missed appointments at 3‐months postoperatively

Political challenges

- Absence of a national support policy for prevention and treatment and for long‐term monitoring of women with fistula

While the MSO program presents numerous successes, certain challenges remain—most notably that of resource limitations, including inconsistent funding, lack of trained healthcare providers, and an inadequate medical supply chain. The MSO team cites an ongoing dilemma: providers frequently diagnose other important gynecologic conditions (i.e. pelvic organ prolapse, urinary incontinence), yet lack the resources to provide treatment for these conditions owing to mandated funding for fistula only. While support for fistula surgery is essential, the need for comprehensive obstetric and gynecologic care for Congolese women remains. Similarly, family planning methods are not readily available for women who have undergone surgical repair, which may complicate postoperative healing.

The MSO program demonstrates to women and their communities that fistula is both preventable and curable and dispels local perceptions that it is a result of sorcery, witchcraft, or a curse. By bringing quality health services to remote villages and subsequently training local healthcare workers, the MSO program addresses health inequities related to poverty, rural living, and access to skilled providers. For these reasons, we believe this program represents a model of care that may be implemented on a national level to definitively address the severe burden of this condition on women, their families, and communities.

The MSO model aims to build capacity to address fistula by improving accessibility to skilled medico‐surgical care and enhancing community awareness. Training efforts serve to improve skills of local providers, strengthen the health workforce, and offer scalable, sustainable solutions to prevention and treatment efforts. Future work will emphasize outcomes research related to patient care delivery, training, and capacity building.

## AUTHOR CONTRIBUTIONS

RM, LK, AN, and DM conceptualized the paper and wrote the original draft. RM, LK, LH, and DM developed the methodology. All authors reviewed and edited the final manuscript.

## CONFLICTS OF INTEREST

The authors have no conflicts of interest.

## References

[1] Hillary CJ , Osman NI , Hilton P , Chapple CR . The aetiology, treatment, and outcome of urogenital fistulae managed in well‐and low‐resourced countries: A systematic review. Eur Urol. 2016;70:478–492. 26922407 10.1016/j.eururo.2016.02.015

[2] Mukwege D , Berg M . A holistic, person‐centred care model for victims of sexual violence in Democratic Republic of Congo: The Panzi Hospital One‐Stop Centre Model of Care. PLoS Med. 2016;13:e1002156. 27727282 10.1371/journal.pmed.1002156 PMC5058554

[3] Longombe AO , Claude KM , Ruminjo J . Fistula and traumatic genital injury from sexual violence in a conflict setting in Eastern Congo: Case studies. Reprod Health Matters. 2008;16:132–141. 18513615 10.1016/S0968-8080(08)31350-0

[4] Watt M , Dennis A , Wilson S , et al. Experiences of social support among women presenting for obstetric fistula repair surgery in Tanzania. Int J Womens Health. 2016;8:429–439. 27660492 10.2147/IJWH.S110202 PMC5019876

[5] Fistula Care . Living with Obstetric Fistula: Qualitative Findings from Bangladesh and the Democratic Republic of Congo. New York, NY: EngenderHealth; 2012 https://www.fistulacare.org/wp-fcp/wp-content/uploads/pdf/technical-briefs/qualitative_fistula_brief_final_web8.13.2012.pdf. Accessed July 8, 2019.

[6] Ahmed S , Holtz SA . Social and economic consequences of obstetric fistula: Life changed forever? Int J Gynecol Obstet. 2007;99(Suppl.1):S10–S15. 10.1016/j.ijgo.2007.06.011 17727854

[7] UNICEF . DRC in 2018 https://www.unicef.org/drcongo/media/2521/file/UNICEF%20RDC%20en%202018.pdf. Accessed July 8, 2019.

[8] Onsrud M , Sjøveian S , Mukwege D . Cesarean delivery‐related fistulae in the Democratic Republic of Congo. Int J Gynecol Obstet. 2011;114:10–14. 10.1016/j.ijgo.2011.01.018 21529808

[9] The World Bank . The World Bank in DRC. Overview. http://www.worldbank.org/en/country/drc/overview. Accessed July 8, 2019.

[10] Ministry of Health of the Democratic Republic of Congo . National strategic plan for the elimination of obstetric fistula [In French]. Kinshasa, DRC, 2018.

[11] UNFPA . End the tragedy of obstetric fistula in DR Congo. 2016 https://drc.unfpa.org/fr/news/en-finir-avec-la-trag%C3%A9die-de-la-fistule-obst%C3%A9tricale-en-rd-congo. Accessed July 8, 2019.

[12] Mukwege D , Amisi C . Panzi Hospital Annual Report 2017. Bukavu, DRC.

[13] Mukwege D , Peters L , Amisi C , Mukwege A , Smith AR , Miller JM . Panzi score as a parsimonious indicator of urogenital fistula severity derived from Goh and Waaldijk classifications. Int J Gynecol Obstet. 2018;142:187–193. 10.1002/ijgo.12514 PMC6436824 29705989

